# Supplementary material for: Modeling glioblastoma heterogeneity as a dynamic network of cell states
Source: Mol Syst Biol. 2021 Sep 16;17(9):e10105. doi: 10.15252/msb.202010105 (PMC8444284; doi:10.15252/msb.202010105)
Supplement: Supplementary file 5 — Source Data for Figure 3 [file MSB-17-e10105-s001.zip › Figure3A_sourcedata/GSEA_3065/nosigdb_state2.GseaPreranked.1622718419215/LARSSON_STATE_3.html]

Details for gene set LARSSON\_STATE\_3[GSEA]

|  || Dataset | state2 |
| Phenotype | NoPhenotypeAvailable |
| Upregulated in class | na\_neg |
| GeneSet | LARSSON\_STATE\_3 |
| Enrichment Score (ES) | -0.76481247 |
| Normalized Enrichment Score (NES) | -2.8915637 |
| Nominal p-value | 0.0 |
| FDR q-value | 0.0 |
| FWER p-Value | 0.0 |
Table: GSEA Results Summary

  

Fig 1: Enrichment plot: LARSSON\_STATE\_3      
 Profile of the Running ES Score & Positions of GeneSet Members on the Rank Ordered List

  

| PROBE | GENE SYMBOL | GENE\_TITLE | RANK IN GENE LIST | RANK METRIC SCORE | RUNNING ES | CORE ENRICHMENT || 1 | TOP2A |  |  | 15 | 0.596 | 0.0295 | No |
| 2 | MKI67 |  |  | 17 | 0.590 | 0.0603 | No |
| 3 | CENPF |  |  | 34 | 0.518 | 0.0857 | No |
| 4 | ASPM |  |  | 122 | 0.342 | 0.0943 | No |
| 5 | NASP |  |  | 156 | 0.306 | 0.1067 | No |
| 6 | HSP90AA1 |  |  | 600 | 0.164 | 0.0679 | No |
| 7 | SYNE2 |  |  | 853 | 0.132 | 0.0479 | No |
| 8 | HNRNPU |  |  | 1369 | 0.094 | -0.0022 | No |
| 9 | GGH |  |  | 2149 | 0.059 | -0.0824 | No |
| 10 | PRKDC |  |  | 2154 | 0.059 | -0.0797 | No |
| 11 | MYH10 |  |  | 3132 | 0.031 | -0.1825 | No |
| 12 | TFPI2 |  |  | 3215 | 0.029 | -0.1898 | No |
| 13 | HSP90AB1 |  |  | 4656 | 0.005 | -0.3435 | No |
| 14 | PIF1 |  |  | 5869 | -0.014 | -0.4723 | No |
| 15 | FUS |  |  | 6216 | -0.020 | -0.5082 | No |
| 16 | MANF |  |  | 7013 | -0.037 | -0.5914 | No |
| 17 | CD320 |  |  | 7342 | -0.046 | -0.6240 | No |
| 18 | LAPTM4B |  |  | 7427 | -0.048 | -0.6305 | No |
| 19 | VGF |  |  | 8046 | -0.069 | -0.6930 | No |
| 20 | LPL |  |  | 8087 | -0.071 | -0.6935 | No |
| 21 | RPN2 |  |  | 8384 | -0.088 | -0.7206 | No |
| 22 | SPCS3 |  |  | 8396 | -0.089 | -0.7171 | No |
| 23 | NCAM1 |  |  | 8660 | -0.108 | -0.7396 | No |
| 24 | MMP16 |  |  | 8897 | -0.135 | -0.7577 | Yes |
| 25 | EMP3 |  |  | 8918 | -0.137 | -0.7527 | Yes |
| 26 | RTN3 |  |  | 8926 | -0.138 | -0.7463 | Yes |
| 27 | DSEL |  |  | 8943 | -0.140 | -0.7406 | Yes |
| 28 | PGRMC1 |  |  | 8958 | -0.143 | -0.7347 | Yes |
| 29 | FGFBP3 |  |  | 8960 | -0.143 | -0.7273 | Yes |
| 30 | IQGAP1 |  |  | 8965 | -0.143 | -0.7203 | Yes |
| 31 | CCDC47 |  |  | 8988 | -0.147 | -0.7149 | Yes |
| 32 | PTN |  |  | 8989 | -0.147 | -0.7072 | Yes |
| 33 | PON2 |  |  | 9030 | -0.154 | -0.7034 | Yes |
| 34 | PLD5 |  |  | 9044 | -0.157 | -0.6966 | Yes |
| 35 | SLC44A1 |  |  | 9045 | -0.157 | -0.6884 | Yes |
| 36 | EMC7 |  |  | 9075 | -0.162 | -0.6830 | Yes |
| 37 | STT3B |  |  | 9091 | -0.164 | -0.6761 | Yes |
| 38 | HACD3 |  |  | 9095 | -0.165 | -0.6677 | Yes |
| 39 | TM9SF3 |  |  | 9103 | -0.167 | -0.6598 | Yes |
| 40 | SPTBN1 |  |  | 9111 | -0.169 | -0.6517 | Yes |
| 41 | ALCAM |  |  | 9132 | -0.173 | -0.6448 | Yes |
| 42 | LRPAP1 |  |  | 9144 | -0.176 | -0.6368 | Yes |
| 43 | PLAT |  |  | 9145 | -0.176 | -0.6275 | Yes |
| 44 | SPCS2 |  |  | 9149 | -0.177 | -0.6186 | Yes |
| 45 | PRDX4 |  |  | 9151 | -0.178 | -0.6094 | Yes |
| 46 | PDIA6 |  |  | 9165 | -0.180 | -0.6014 | Yes |
| 47 | WLS |  |  | 9168 | -0.181 | -0.5922 | Yes |
| 48 | HMGCR |  |  | 9194 | -0.187 | -0.5850 | Yes |
| 49 | SCRG1 |  |  | 9200 | -0.191 | -0.5756 | Yes |
| 50 | SOX6 |  |  | 9203 | -0.192 | -0.5658 | Yes |
| 51 | FAT1 |  |  | 9207 | -0.193 | -0.5560 | Yes |
| 52 | SQLE |  |  | 9209 | -0.194 | -0.5459 | Yes |
| 53 | MDM4 |  |  | 9211 | -0.197 | -0.5357 | Yes |
| 54 | LMAN1 |  |  | 9216 | -0.199 | -0.5258 | Yes |
| 55 | GRIA2 |  |  | 9222 | -0.202 | -0.5157 | Yes |
| 56 | DYNC1H1 |  |  | 9231 | -0.205 | -0.5059 | Yes |
| 57 | ABHD2 |  |  | 9250 | -0.213 | -0.4967 | Yes |
| 58 | TMBIM6 |  |  | 9264 | -0.219 | -0.4866 | Yes |
| 59 | CALU |  |  | 9278 | -0.223 | -0.4763 | Yes |
| 60 | LAMC1 |  |  | 9286 | -0.226 | -0.4652 | Yes |
| 61 | GPNMB |  |  | 9290 | -0.228 | -0.4536 | Yes |
| 62 | PDIA3 |  |  | 9297 | -0.230 | -0.4423 | Yes |
| 63 | TMED9 |  |  | 9305 | -0.233 | -0.4308 | Yes |
| 64 | CALR |  |  | 9310 | -0.237 | -0.4189 | Yes |
| 65 | CD9 |  |  | 9324 | -0.248 | -0.4073 | Yes |
| 66 | GPC1 |  |  | 9326 | -0.250 | -0.3943 | Yes |
| 67 | BSG |  |  | 9331 | -0.253 | -0.3815 | Yes |
| 68 | HSP90B1 |  |  | 9334 | -0.257 | -0.3683 | Yes |
| 69 | PTPRZ1 |  |  | 9351 | -0.272 | -0.3558 | Yes |
| 70 | BCAN |  |  | 9353 | -0.276 | -0.3415 | Yes |
| 71 | SSR4 |  |  | 9359 | -0.287 | -0.3270 | Yes |
| 72 | ATP6AP2 |  |  | 9365 | -0.294 | -0.3121 | Yes |
| 73 | CANX |  |  | 9374 | -0.310 | -0.2968 | Yes |
| 74 | GPM6A |  |  | 9381 | -0.324 | -0.2804 | Yes |
| 75 | B2M |  |  | 9383 | -0.331 | -0.2632 | Yes |
| 76 | TIMP1 |  |  | 9387 | -0.334 | -0.2461 | Yes |
| 77 | CD63 |  |  | 9394 | -0.353 | -0.2282 | Yes |
| 78 | ITM2B |  |  | 9399 | -0.371 | -0.2093 | Yes |
| 79 | HSPA5 |  |  | 9406 | -0.395 | -0.1892 | Yes |
| 80 | APLP2 |  |  | 9409 | -0.408 | -0.1681 | Yes |
| 81 | APP |  |  | 9412 | -0.426 | -0.1461 | Yes |
| 82 | KCNQ1OT1 |  |  | 9414 | -0.434 | -0.1235 | Yes |
| 83 | MTRNR2L12 |  |  | 9416 | -0.436 | -0.1008 | Yes |
| 84 | DST |  |  | 9422 | -0.485 | -0.0760 | Yes |
| 85 | MALAT1 |  |  | 9436 | -0.735 | -0.0389 | Yes |
| 86 | POSTN |  |  | 9437 | -0.750 | 0.0003 | Yes |
Table: GSEA details [plain text format]

  

Fig 2: LARSSON\_STATE\_3: Random ES distribution      
 Gene set null distribution of ES for **LARSSON\_STATE\_3**
